# Case Report: Intracranial ectopic schwannoma originating from the internal carotid artery wall

**DOI:** 10.3389/fsurg.2025.1475191

**Published:** 2025-02-13

**Authors:** Zhige Guo, Wahafu Alafate, Wu Gan

**Affiliations:** Department of Neurosurgery, Guangdong Provincial People’s Hospital (Guangdong Academy of Medical Sciences), Southern Medical University, Guangzhou, Guangdong, China

**Keywords:** ectopic schwannoma, internal carotid artery wall, good prognosis, gross total removal of tumor, case report

## Abstract

This case report presents a rare instance of an ectopic schwannoma originating from the internal carotid artery wall in a 21-year-old male, initially misdiagnosed as an anterior clinoid meningioma. The patient presented with intermittent headaches and dizziness for over two years, aggravated for the past month. Brain MRI revealed a mass above the left side of the parasellar region, hypointense on T1-weighted images and heterogeneously hyperintense on T2-weighted and T2 FLAIR sequences. The operation involved a left frontotemporal craniotomy, and the tumor was found to be compressing and displacing the ipsilateral optic nerve and internal carotid artery. The tumor was completely resected, and postoperative MRI confirmed no residual tissue. Histopathological examination confirmed the diagnosis of schwannoma. This case is unique in its origin from the internal carotid artery wall and its favorable prognosis, with complete functional recovery and resolution of symptoms. The report emphasizes the importance of careful surgical approach and the excellent prognosis of paraclinoid region schwannomas.

## Introduction

Schwannomas are common tumors of cranial and spinal nerve Schwann cells, accounting for approximately 8%–10% of intracranial tumors (1). They are frequently observed in forms such as acoustic neuromas and trigeminal schwannomas. Intracranial schwannomas without a clear origin from cranial nerves are referred to as ectopic schwannomas (2), including those located within brain parenchyma, intraventricular schwannomas, and parasellar region schwannomas.

A literature search has revealed a total of 40 reported cases of sellar region schwannoma (2), with only one case identified as originating from the internal carotid artery wall, which led to profuse hemorrhage and sacriﬁce of the carotid artery (3). Herein, we presented a rare case of ectopic schwannoma of the parasellar region originating from the internal carotid artery wall with the successful preservation of surrounding normal structures and complete tumor resection.

## Case description

**Figure 1:**
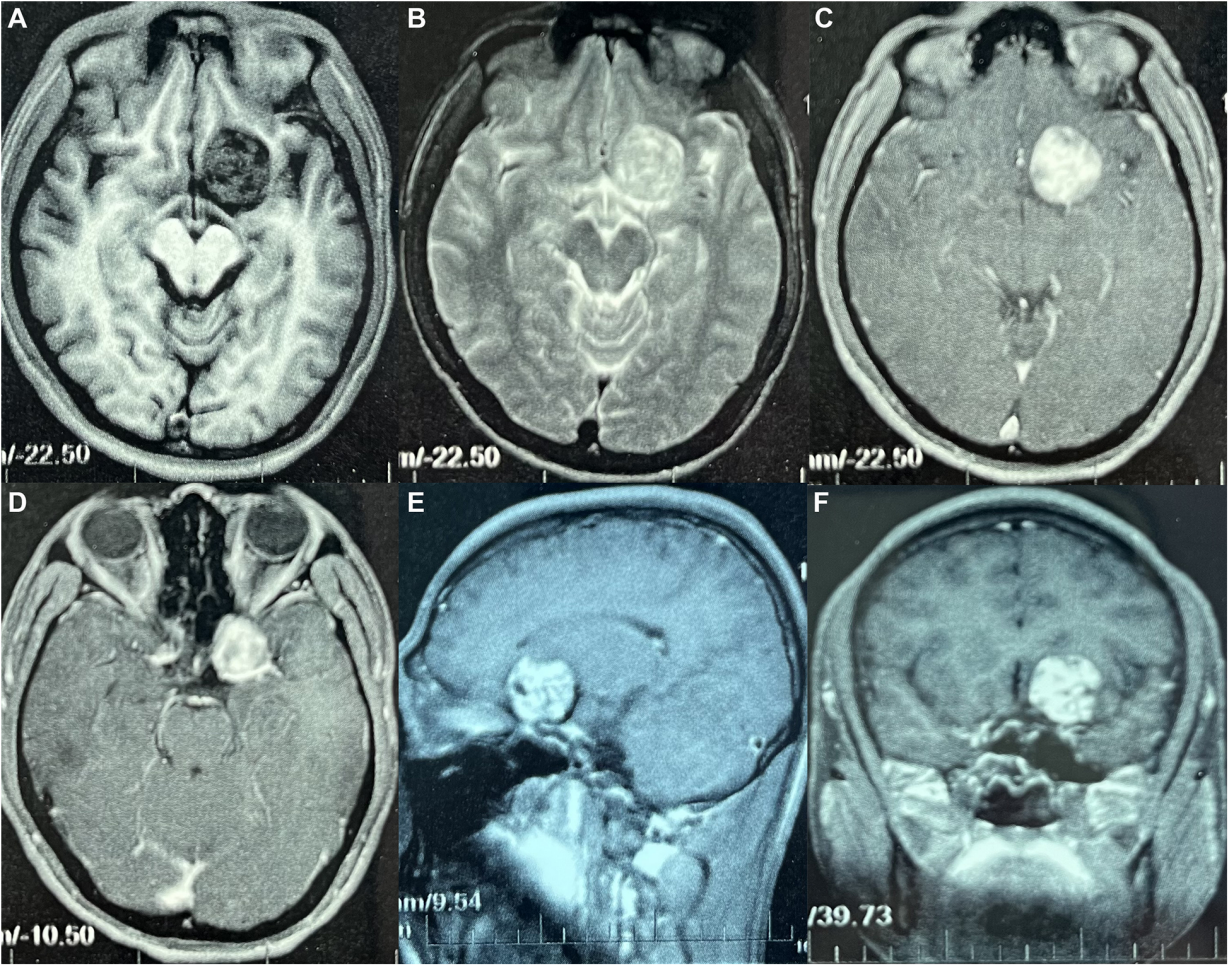
Preoperative magnetic resonance image: **(A)** T1-weighted image (axial section) revealed a hypointense mass above the left side of the sellar region. **(B)** T2-weighted image (coronal section) demonstrates heterogeneously hyperintense signal of the lesion. **(C–F)** Contrast-enhanced T1-weighted image **(C,D)** axial section; **(E)** sagittal section; **(F)** coronal section, showing homogenous enhancement of the lesion.

A 21-year-old male was first admitted on July 15, 2023, with a history of “intermittent headaches and dizziness for over 2 years, aggravated for over 1 month”, with no previous medical history of schwannomas or family history of neurofibromatosis. Physical examination of the patient revealed clear consciousness and intact orientation. The twelve cranial nerves were normal upon examination. Muscle tone in all four limbs was within normal limits, with muscle strength rated at grade 5. Both superficial and deep reflexes were normal. Pathological reflexes and meningeal irritation signs were negative. Brain MRI (Figure 1) revealed a mass located superior to the left side of the parasellar region, approximately 30 mm × 29 mm × 31 mm in size, with well-defined margins. The lesion appeared hypointense on T1-weighted images, heterogeneous hyperintense on T2-weighted and T2 Fluid Attenuated Inversion Recovery (FLAIR) sequences, hypointense on Diffusion-Weighted Imaging (DWI), with no significant surrounding edema. Contrast enhancement was uniform and consistent, with no obvious meningeal tail sign. The preoperative diagnosis is Anterior Clinoid Meningioma.

## Diagnostic assessment

### Operation

**Figure 2:**
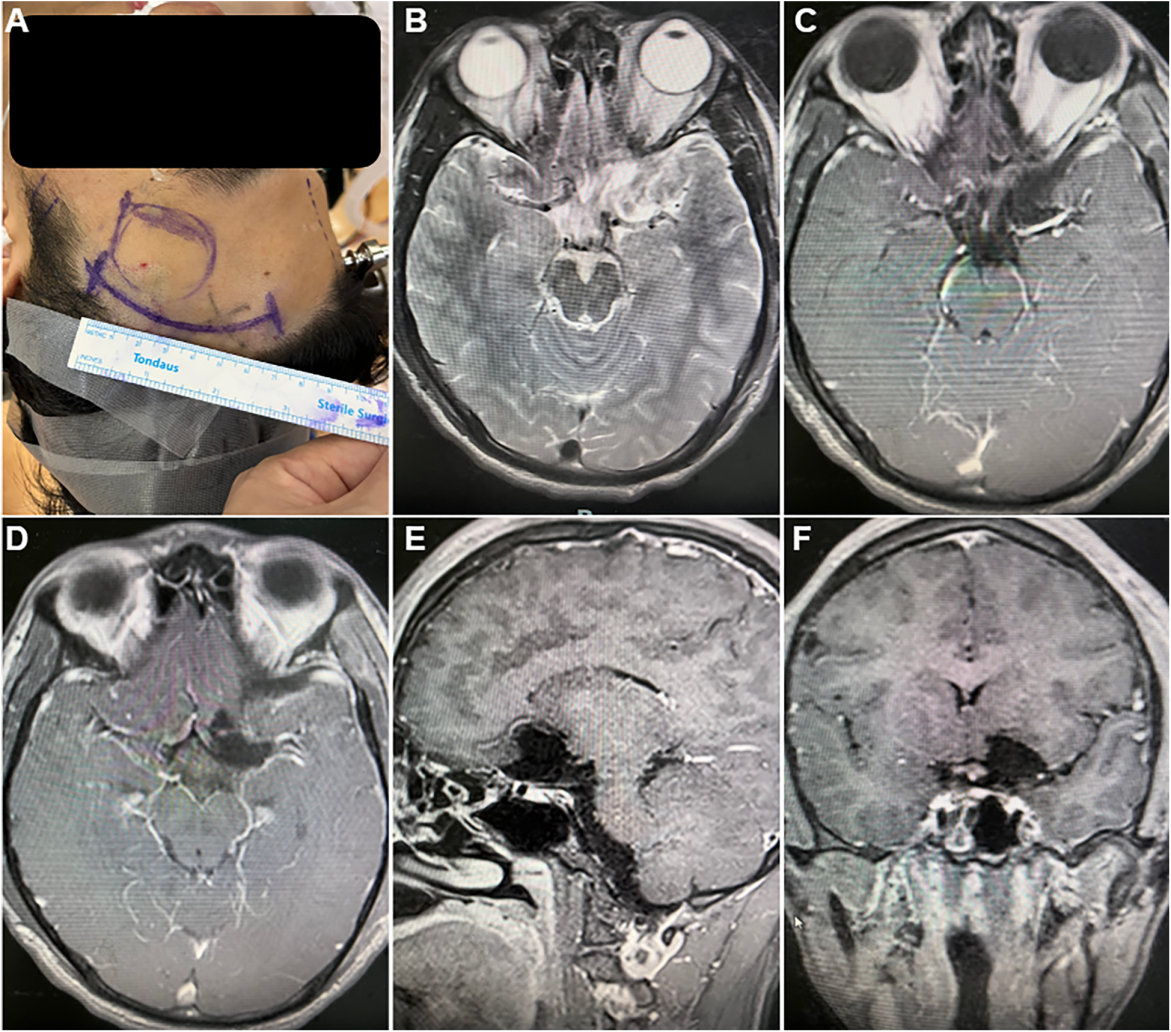
Surgical incision and postoperative magnetic resonance image. **(A)** the curvilinear surgical incision within the left frontotemporal hairline. **(B–F)** postoperative magnetic resonance image revealed complete resection of the tumor, with no residual tissue, hemorrhage, or fluid accumulation: **(B)** T2-weighted image (axial section); **(C–F)** Contrast-enhanced T1-weighted image **(C,D)** axial section; **(E)** sagittal section; **(F)** coronal section.

**Figure 3:**
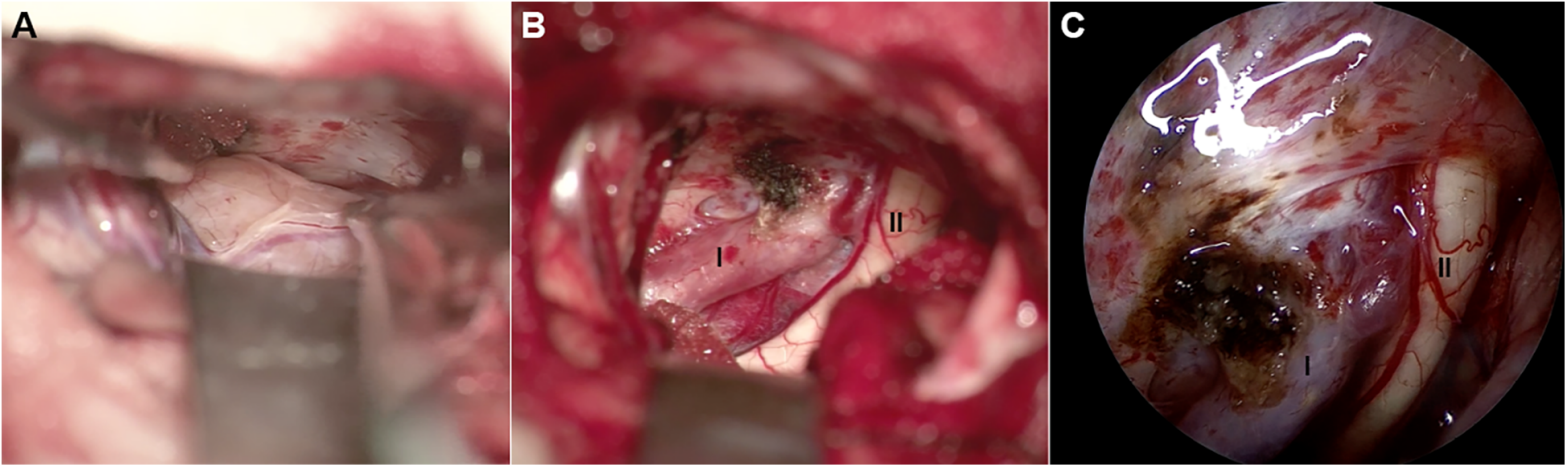
Intraoperative picture. **(A,B)** Microscopy view and **(C)** neuroendoscope view of surgical field. **(A)** The pale yellow, medium soft in texture, and well-vascularized ectopic schwannoma is exposed. **(B,C)** The tumor is completely excised, internal carotid artery (I) and ipsilateral optic nerve (II) are clearly revealed.

A curvilinear incision was made within the left frontotemporal hairline (Figure 2). Upon opening, the temporal muscle was dissected and retracted. A craniotomy of approximately 3 cm × 2.5 cm was performed using a craniotome and milling drill. Some bone tissue in the direction of the anterior cranial base was removed with a burr drill. After hemostasis was achieved by elevating the dura mater, the dura was incised. Under the microscope, the sylvian fissure was opened, cerebrospinal fluid was released, and the frontal lobe was retracted to expose the tumor tissue, which was pale yellow, medium soft in texture, and well-vascularized. Exploration revealed compression and displacement of the ipsilateral optic nerve and internal carotid artery. The tumor base was located at the anterior internal carotid artery, partially involving the lateral wall of the cavernous sinus. Initially, hemostasis was performed at the tumor base, and the tumor's blood supply was disconnected. Most of the tumor was then removed intratumorally. Subsequently, the surrounding interface was separated, with slight adhesions, and the tumor was completely resected after freeing it. A 30° neuroendoscope was inserted into the surgical area for further exploration. The tumor base was well managed, surrounding structures were clearly protected, and no significant residual tumor was observed (Figure 3).

## Result

Postoperative follow-up brain MRI revealed complete resection of the tumor, with no residual tissue, hemorrhage, or fluid accumulation (Figure 3). The patient exhibited no neurological deficits and was discharged on the third postoperative day. Six months later, a follow-up examination indicated a Karnofsky Performance Status (KPS) score of 100, with complete functional recovery and overall good condition. Symptoms of headache and dizziness had resolved.

### Pathology

**Figure 4:**
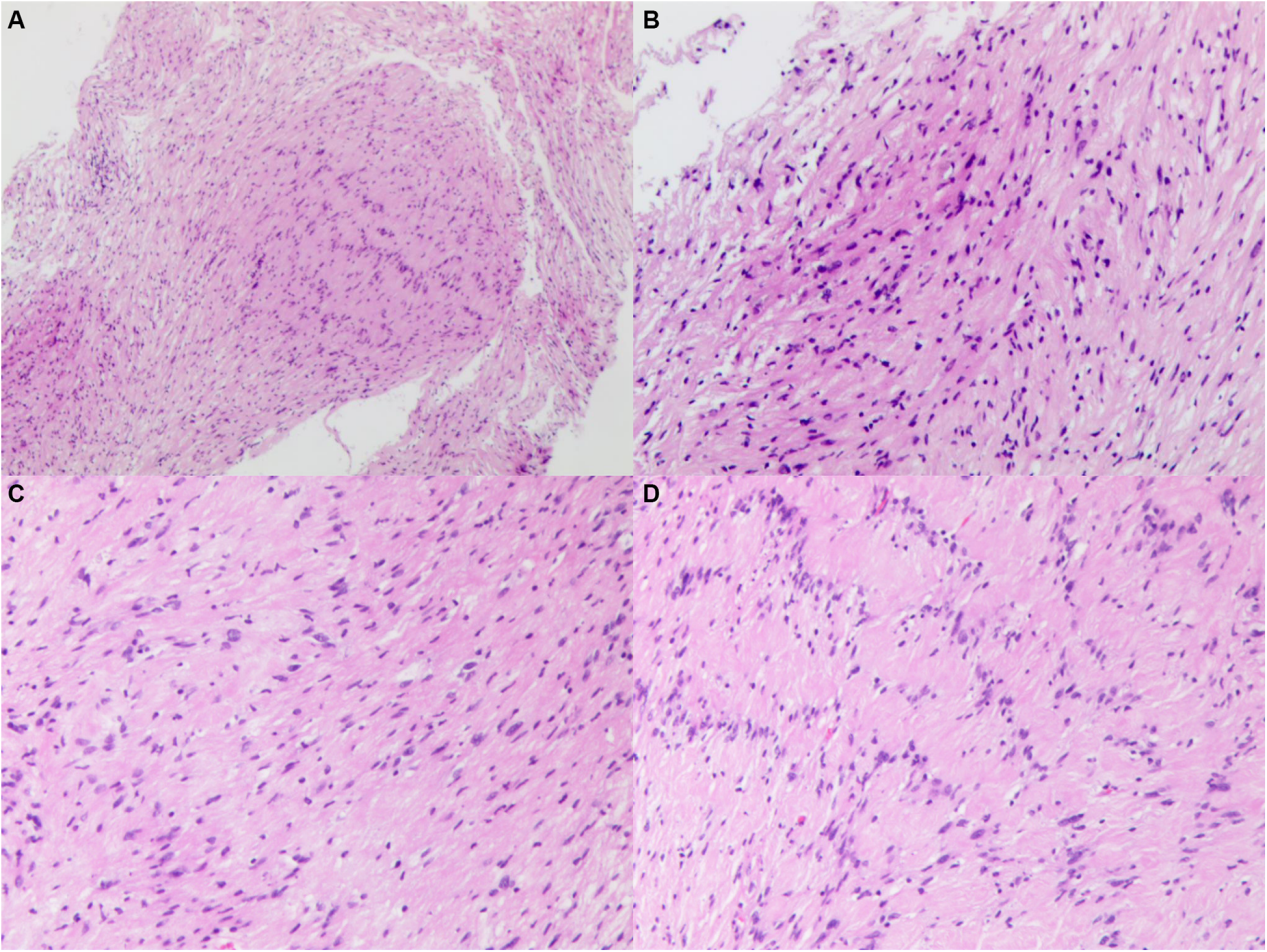
Histopathological image of specimen, showing tumor cells with a spindle shape, abundant cytoplasm, and rod-shaped or wavy nuclei, arranged in a palisading and whorled pattern with variable density. The interstitial vessels were dilated and congested, accompanied by hyalinization. **(A)** original magnification ×40. **(B–D)** Original magnification ×100.

Microscopic examination of the pathological specimen revealed tumor cells with a spindle shape, abundant cytoplasm, and rod-shaped or wavy nuclei, arranged in a palisading and whorled pattern with variable density. The interstitial vessels were dilated and congested, accompanied by hyalinization. Immunohistochemical staining showed the tumor cells to be negative for Epithelial Membrane Antigen (EMA), positive for Vimentin (++), D2-40 (++), and negative for Progesterone Receptor (PR). O—6—Methylguanine—DNA Methyltransferase (MGMT) expression was strong (+++), while Somatostatin Receptor 2 (SSTR2) was negative. S100 and SRY—box 10 (SOX10) were positively expressed (+++), and Protein gene product 9.5 (PgP9.5) was also strongly positive (+++). Neurofilament (NF), Glial Fibrillary Acidic Protein (GFAP), and Oligodendrocyte Transcription Factor 2 (Olig2) were negative, and the Ki67 index was approximately 5% positive in hot spots. Special staining with silver (reticulin fiber staining) was positive. The pathological diagnosis was schwannoma (Figure 4). For the pathological diagnosis of schwannoma, positive S100 and SOX10 are necessary diagnostic criteria. Meanwhile, some pathological markers are of great significance in differentiating schwannoma from other tumors: EMA for differentiating from epithelial-derived tumors, NF for differentiating from neuron-derived tumors, GFAP and Olig2 for differentiating from glial cell-derived tumors, and D2-40 for differentiating from lymphoma.

## Discussion

Ectopic schwannomas are a particularly rare occurrence. In known cases, these tumors predominantly affect elder individuals, with an average age of 49.5 years (range: 19–79 years). The incidence is fairly equal between males and females (20:19) (2). The diagnosis of sellar ectopic schwannomas can be challenging, as they are often misdiagnosed as pituitary adenomas. These tumors pose higher surgical risks due to their highly vascular nature. Among a total of 40 reported cases of sellar region schwannoma (2), only one case was identified as originated from the internal carotid artery wall: a 57-year-old female with a 1.9 cm intrasellar-suprasellar tumor, initially considered to be an anterior clinoid meningioma. During surgery, the tumor was found to be tightly adhered to the internal carotid artery wall, difficult to separate, leading to massive hemorrhage and necessitating internal carotid artery embolization and clamping, resulting in sacrifice of the left internal carotid artery. Postoperative complications included cerebral infarction, and the pathological diagnosis was schwannoma (3). According to the literature, our case represents the first reported schwannoma with a favorable prognosis originating from the internal carotid artery wall.

Schwann cells are not components of the central nervous system. Regarding the origin of sellar region schwannomas, four histopathological hypotheses have been proposed (4): (1) Perivascular schwann cells of the nervi vasorum, the perivascular plexuses of the internal carotid artery or the hypophyseal artery are considered potential sources of schwannomas, as cerebral arteries are accompanied by perivascular plexuses where schwann cells can be found (3, 5). In the literature (3) and in the present case, the close adherence between the tumor and the internal carotid artery suggests an origin from the perivascular plexus of the internal carotid artery. (2) Schwann cells of the parasellar plexus of the cavernous sinus, which can account for the development of parasellar schwannomas (6), and schwannomas of the plexus can extend into the sella through potential defects in the medial wall of the cavernous sinus (7). (3) Schwann cells enveloping small nerve branches that innervate the dura mater, with cases in the literature adhering tightly to the dura mater suggesting this as a secondary origin (8). (4) Ectopic schwann cells, located within the sella, are considered the source of intrasellar schwannomas (9).

However, in reality, intracranial supra-sellar sensation, including pain, is primarily innervated by the trigeminal nerve, particularly its ophthalmic branch, which provides extensive neurovascular innervation to the dura mater and major cerebral blood vessels, a recognition that has been established for some time (10, 11). The trigeminal vascular system, located at the interface between the nervous and vascular systems, can effectively detect sensory inputs and influence blood flow regulation. Additionally, studies on awake patients during surgery have demonstrated that mechanical stimulation of the leptomeninges and small cerebral vessels can induce pain (11). Therefore, it is reasonable to believe that the various sources of schwannoma, including perivascular schwann cells of the nervi vasorum, parasellar plexus schwann cells of the cavernous sinus, schwann cells enveloping small nerve branches innervating the dura mater, and so-called ectopic schwann cells, are all terminal branches of the trigeminal nerve distributed on cerebral blood vessels and meninges. Certain stimuli that induce schwann cell proliferation may lead to the development of schwannomas, and this can also explain the occurrence of intraparenchymal and intraventricular schwannomas. In this case, the patient experienced trigeminal-related headache symptoms preoperatively, which gradually resolved after tumor resection, indirectly indicating the correlation between the tumor and the trigeminal nerve.

Schwannomas are typically yellow or gray, rich in vascularity, and rubbery in consistency. The partial adherence of some sellar region schwannomas to adjacent structures makes complete resection challenging. The rate of total resection through a transsphenoidal approach is reported to be 36.0%. Literature reports two cases of massive intraoperative hemorrhage and two cases of postoperative cerebral infarction (3). Particularly, when they adhere tightly to the internal carotid artery, aggressive attempts at complete resection should be avoided. Slight residual tumors can be treated with electrocoagulation. Given the benign nature of these tumors, the prognosis is excellent, and no further radiotherapy is required.

## Conclusion

Ectopic schwannomas originating from the internal carotid artery wall are indeed a rarity. This case marks the first reported instance of a favorable prognosis for an ectopic schwannoma arising from the internal carotid artery wall, with the successful preservation of surrounding normal structures and complete tumor resection.

## Data Availability

The original contributions presented in the study are included in the article/Supplementary Material, further inquiries can be directed to the corresponding author.
